# Feasibility of Performing Supine Percutaneous Nephrolithotomy for Solitary Renal Pelvic Stones (1.5-3 cm) Under Spinal Anaesthesia: A Single-Centre Study

**DOI:** 10.7759/cureus.38472

**Published:** 2023-05-03

**Authors:** Rahul Gupta, Suhail M Khan, Sunana Gupta, Arti Mahajan, Chetan Sharma, Chaman Lal Gupta

**Affiliations:** 1 Urology, Government Medical College, Jammu, Jammu, IND; 2 Anesthesia and Critical Care, All India Institute of Medical Sciences, Jammu, Jammu, IND; 3 Anesthesia and Critical Care, Government Medical College, Jammu, Jammu, IND

**Keywords:** solitary pelvic stones, spinal anesthesia, pcnl, pelvic stones, supine

## Abstract

Introduction

Percutaneous nephrolithotomy (PCNL) procedure has evolved over the years and one such evolution has been the introduction of supine PCNL by Valdivia in 1987. This approach offers the added advantage of safe access in patients with compromised cardiovascular and pulmonary function. General anesthesia is the preferred anesthetic technique for PCNL. However, in the last decade, there has been an increase in the usage of regional anesthesia for this procedure. We share our experience of supine PCNL for solitary renal pelvic stones under regional anesthesia.

Aim and objective

To assess the feasibility, safety, and efficacy of supine PCNL for solitary renal pelvic stones sized 1.5 to 3 cm under spinal anesthesia.

Material and methods

This was a retrospective record review of 35 patients (21 male) who underwent supine PCNL under regional anesthesia between January 2022 till December 2022 at our institute. All patients had a solitary renal pelvic calculus sized 1.5-3 cm. Intraoperative and postoperative data were analyzed.

Results

The mean age of patients was 38.5 ± 15 years. The postoperative pain visual analog scale (VAS) score was <5 in 31 (89%) and >5 in 4 (11%) patients. The mean hospital stay was 3.33 ± 0.88 days. Mean fall in hemoglobin level was 0.49 ± 0.43 mg/dL. Postoperatively, mild hematuria occurred in three patients (8.5%) and fever occurred in two (5.7%) patients. There was no injury to adjacent organs in this series. Blood transfusion was required only in one patient. Complete stone clearance was achieved in all patients.

Conclusion

In experienced hands, supine PCNL under regional anesthesia is a feasible, safe, and effective approach with minimal morbidity.

## Introduction

Percutaneous nephrolithotomy (PCNL) was first described in 1976 and it has since gained popularity and is now the procedure of choice for renal calculus sized >2 cm. PCNL was originally performed in the prone position [1-4]. This position was selected based on the anatomical considerations related to the posterior retroperitoneal location of the kidneys. The prone position provides convenient access to the posterior calyces located on the avascular line of Brodel, providing substantial surface area for puncture. Moreover, this position reduces the risk of interposition of other viscera along the working tract [5]. Thus, prone PCNL was the standard procedure and the only approach to access the pelvicalyceal system until Valdivia et al. (1987) described the performance of PCNL in the supine position [6].

The aim of choosing the supine position was to circumvent the patient-, anesthesia-, and surgery-related problems associated with the prone position. The supine position confers several benefits over prone position, including better patient comfort, lower risk of cardiopulmonary complications, no requirement for patient repositioning, and minimal radiation exposure for the operator. Moreover, supine PCNL affords convenient and direct access to the anesthesiologist in case of any medical emergency. It provides a more ergonomic setting for the urologist and, if necessary, allows for an easier endoscopic combined intrarenal surgery (ECIRS) approach [7].

PCNL is commonly performed under general anesthesia (GA). This enables better hemodynamic and airway control and alleviates patient discomfort caused by prone positioning [8]. However, patients with pulmonary atelectasis, chronic obstructive pulmonary disease, and spinal cord injury are not suitable for GA. Hu et al. performed a meta-analysis of studies comparing the use of regional anesthesia (RA) versus GA for PCNL. They reported that patients in the RA group experienced less discomfort on the first postoperative day than those in the GA group (P=0.005). Moreover, the mean operation time (P=0.005) and length of hospital stay were shorter in the RA group [8]. In terms of stone-free status, the type of anesthesia had no impact on clearance rates. GA also adds to the overall cost of the procedure [9].

There is a paucity of data on the outcomes of PCNL performed in the supine position under spinal anesthesia. A recent study by Gupta et al. demonstrated the technical feasibility of supine PCNL and showed that it can be performed under general and spinal anesthesia in carefully selected patients [10].

Based on our experience of performing prone PCNL under RA, we started supine PCNL also under RA. In this study, we retrospectively investigated the safety and efficacy of supine PCNL performed under RA for solitary renal pelvic stones of up to 3 cm.

## Materials and methods

This was a single-center retrospective study of patients who had undergone supine PCNL for solitary renal pelvic stones of size 1.5-3 cm under spinal anesthesia by a single surgeon. Data were collected and analyzed as per the predetermined proforma.

Study site

The study was conducted at a tertiary care center and the data were collected retrospectively from January 2022 to December 2022. Medical record files of all patients who had undergone supine PCNL for solitary renal pelvic stone were retrieved. Institutional ethics committee clearance was obtained prior to initiating the study. All patient data were anonymized and confidentiality of data was ensured by storing the data in a password-protected file accessible only to the investigators.

Inclusion criteria

1) Adult patients (both male and female; age-range 18-70 years) with solitary renal pelvic calculi of size 1.5-3 cm; 2) normal renal function and sterile urine culture; 3) American Society of Anesthesiologists Physical Status (ASA PS): I, II.

Exclusion criteria

1) Patients with renal anomalies (e.g., horseshoe kidney, ectopic kidney); 2) presence of multiple stones/staghorn stones; 3) patients with an un-correctable coagulopathy; 4) pregnant women; 5) any contraindication for RA; 6) active urinary tract infections.

Pre-operative work-up

This included a detailed history and physical examination including the standard urological assessment for all patients with renal stones. All baseline investigations along with coagulation profile and radiological investigations (conventional intravenous urography/computed tomography-intravenous pyelography [CT-IVP]) were performed. As per the protocol in our unit, all patients had sterile urine before performing supine PCNL. Any comorbidity was recorded in the proforma.

Operative procedure

**Figure 1:**
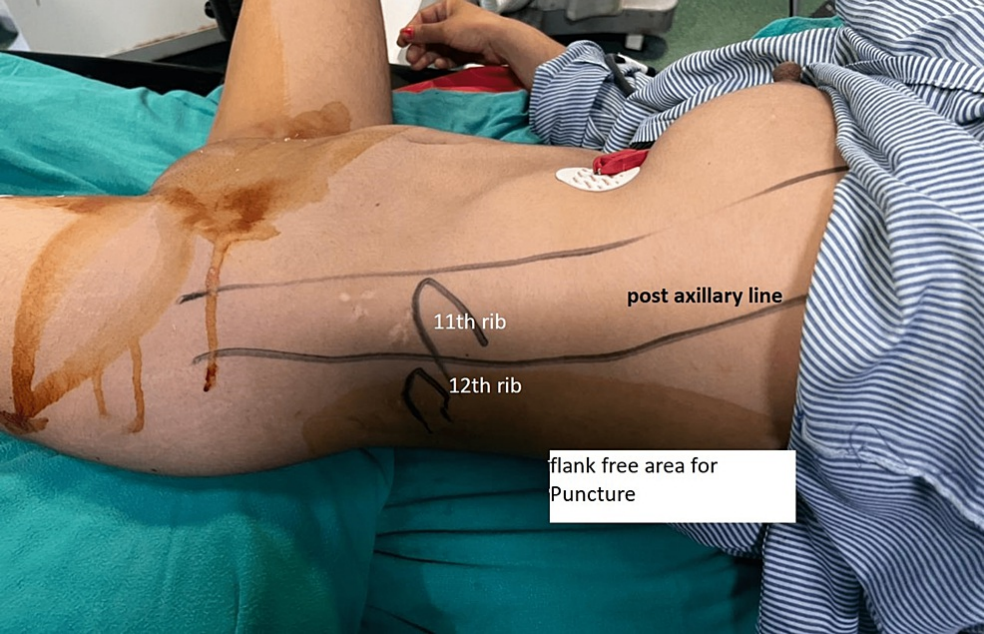
Representative photograph showing the position of the patient with important landmarks marked by a sterile marker. The landmarks include the 12th and 11th ribs, iliac crest, and posterior axillary lines. The surface marking was done in the sitting position.

**Figure 2:**
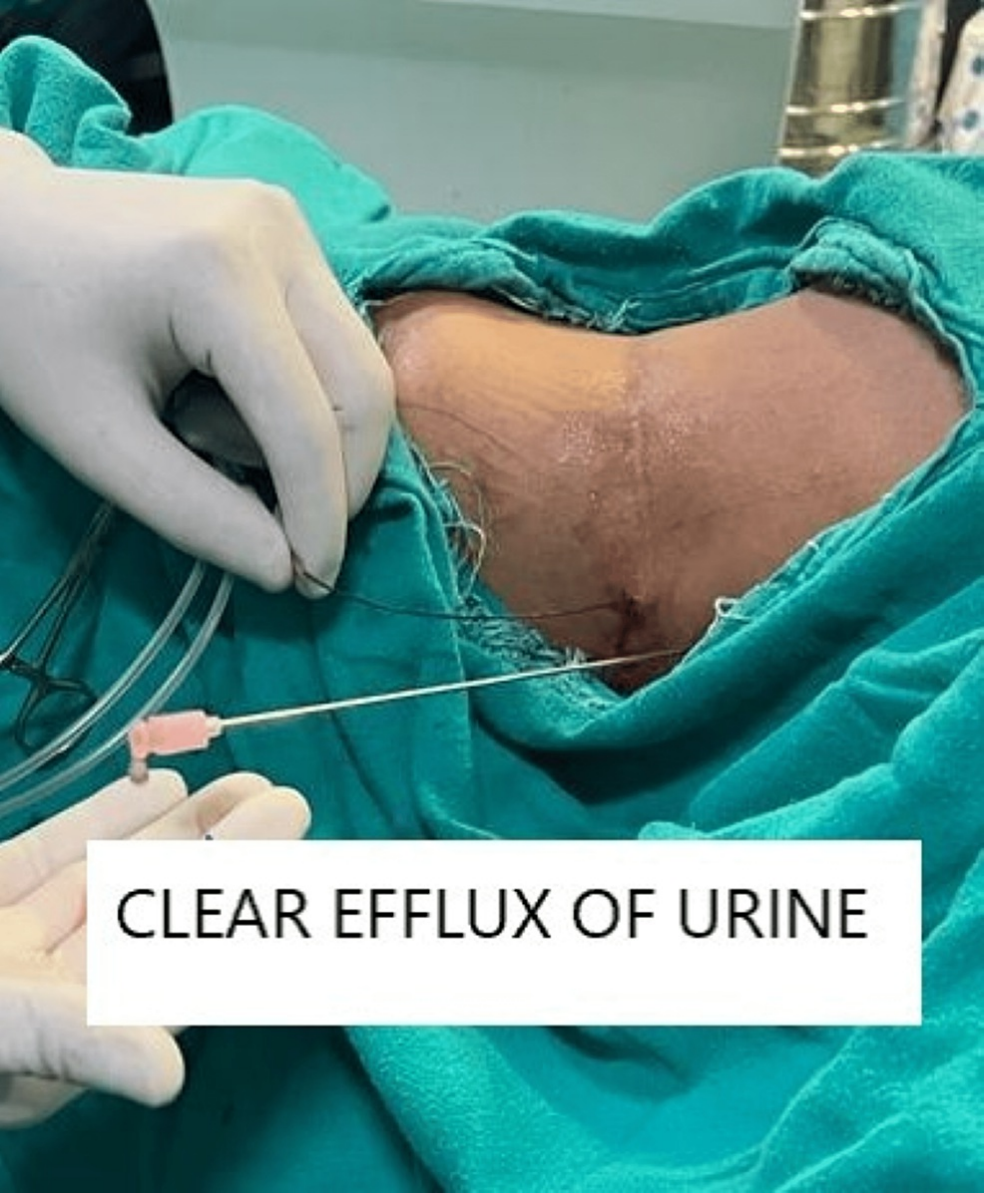
Representative intraoperative photograph showing the clear efflux of urine from the puncture.

Before starting the procedure, the important surface anatomical landmarks (including the 12th and 11th ribs, iliac crest, and, posterior axillary line) were marked by a sterile marker, with the patient in the sitting position (Figure 1). All patients received spinal anesthesia in the lateral position using a 27-gauge spinal needle with 0.5% hyperbaric bupivacaine to achieve a sensory blockade till the T4 level. After placing the patient in the lithotomy position, a 6 Fr ureteral catheter was positioned into the ipsilateral renal collecting system. The patient was then positioned supine in the modified Valdivia Galdakao position (Figure 1). Using the ureteral catheter placed earlier, an air pyelogram was performed to delineate the anatomy of the pelvicalyceal system, and the most suitable calyx was chosen for puncture. The choice of calyx was made on the operating table. An 18 G/20 cm two-part IP-needle (Cook CE, Bloomington, IN, USA) was used to puncture the collecting system under fluoroscopy control and clear egress of the fluid was considered as a precursor to further dilatation of the tract (Figure 2). The C arm position used for puncture was zero degrees and the puncture was confirmed under 30-degree rotation and the clear efflux of the irrigation fluid.

A 0.035-inch straight-tip Terumo guidewire was passed through the puncture needle and placed antegrade into the urinary bladder whenever feasible, and the tract was dilated using polytetrafluoroethylene (PTFE) serial dilators up to 12 Fr. Later, metallic telescopic dilators (Alken's dilators) were used under fluoroscopy and a 20/22 Fr Amplatz was positioned. Using a 19.5 Fr slender Karl Storz nephroscope, the stone was fragmented using either lithotripsy (Swiss LithoClast Master; Electro Medical Systems, Nyon, Switzerland) or HoYAG laser (35 W). Fluoroscopy and visual assessment were done to evaluate the stone-free status at the end of the operation. A 6/26 Fr/cm DJ stent was left in the ureter for a period of three weeks if indicated. A nephrostomy tube of appropriate size was placed in all patients and was removed after 24-48 hours.

The operative time was defined as the time taken from the initial puncture to nephrostomy placement. A plain kidney, ureter, and bladder (KUB) X-ray was obtained prior to nephrostomy removal. Intraoperative parameters recorded were duration of surgery (min), X-ray exposure time (s), distribution of calyceal puncture, number of punctures required, and intraoperative bleeding (hampering vision or leading to abandonment of the procedure). The number of patients who were converted to GA due to inadequate block or any other complication was also noted.

Postoperative parameters recorded were stone clearance rate, postoperative hematuria, drop in hemoglobin levels, blood transfusion rate, visual analog scale (VAS) score, hospital stay, fever and any other complications (as per the Clavien-Dindo classification). Stone clearance was documented by postoperative radiographs and ultrasonography. Hemoglobin level was checked on the 1st postoperative day and was compared with the preoperative level. VAS scores were recorded postoperatively at 12 hrs and 24 hrs using a 10-point scale (0 represented no pain and 10 represented the worst imaginable pain). Patients were asked to indicate the point on the scale based on the severity of the pain.

All patients were followed for three months after the discharge with an ultrasound KUB and urine routine.

## Results

A total of 35 patients (21 [60%] male and 14 [40%] female) were included in this study. The mean age of patients was 38.5 ± 15 years (range, 18-58). The majority of the patients in our study were in their third decade of life. The mean size of the stone in this series was 2.43 ± 0.5 cm (range, 1.5-3). The mean body mass index (BMI) was 27.2 ± 3.2 kg/m^2^. Twenty (57.1%) patients belonged to ASA PS class I and 15 ( 42.8%) patients belonged to ASA PS class II.

The mean operative time was 42 ± 10.3 min and the mean X-ray exposure by C-arm during the procedure was 130 ± 60 s. All patients were dealt with a single puncture. Middle calyx was punctured in 42.8% of cases and inferior calyx was punctured in 40% of cases; 17.14% required superior calyx puncture. The mean number of attempts to access the pelvicalyceal system was 1.6 + 0.67. None of the patients in this series required conversion to GA.

Postoperative pain mean VAS score was <5 in 31 (89%) patients and >5 in four (11%) patients. The mean fall in Hb level after the operation was 0.49 ± 0.43. One patient (2.8%) required blood transfusion in the postoperative period. In this series, three patients (8%) experienced mild hematuria persisting for >48 h which settled with conservative treatment. No injury to adjoining organs occurred in this series. Two patients (5%) developed fever postoperatively which responded to intravenous antibiotics. The mean length of hospital stay was 3.33 ± 0.88 days with a stone clearance rate of 100%. The incidence of complications classified according to the modified Clavien-Dindo grading system is summarized in Table 1.

**Table 1:** Incidence of complications as per the modified Clavien-Dindo grading system

Clavien grade	Description	Number	%
I	Any deviation from the normal postoperative course without the need for pharmacological treatment or surgical, endoscopic, and radiological interventions. Allowed therapeutic regimens are drugs such as antiemetics, antipyretics, analgesics, diuretics, electrolytes and physiotherapy. This grade also includes infections opened at bed site.	3	8
II	Requiring pharmacological treatment with drugs other than such allowed for Grade 1 complications. Blood transfusions and total parental nutrition are also included.	1	2.8
III	Requiring surgical, endoscopic, or radiological intervention	0	0
IIIa	Intervention not under general anesthesia		
IIIb	Intervention under general anesthesia		
IV	Life-threatening complications requiring ICU management.	0	0
IVa	Single organ dysfunction (Dialysis)		
IVb	Multiorgan dysfunction		
V	Death of the patient	0	0

## Discussion

Owing to its minimally invasive nature, percutaneous nephrolithotomy (PCNL) has largely replaced open stone surgery for large and complex renal or upper ureteral calculi [11]. PCNL has been traditionally performed in the prone position. The choice of prone position is largely based on physical and intuitive factors. Over the past few years, PCNL has evolved with the inclusion of new and innovative technologies in terms of various types of nephroscopes and mode of lithotripsy. This has helped improve the safety and effectiveness of procedures [12]. Patient position during PCNL has also undergone a change from prone to supine [6].

The prone position has several limitations. It is relatively contraindicated in morbidly obese patients and those with severe cardiopulmonary disease. Another drawback is that the ureteric catheter must be first inserted in the lithotomy position and then the position needs to be changed to prone. This is a cumbersome undertaking, especially under GA, and the stone can get displaced to an unfavorable position [11].

Supine PCNL was first introduced in 1987 by Gabriel Valdivia [6]. Though supine PCNL is usually performed under GA, it can also be performed under RA [11]. In addition to the well-known advantages of RA over GA, supine PCNL under RA shortens the operative time as there is no need to change the patient position and reversal from neuromuscular blockade. Moreover, the patient can respond to the verbal commands of the operating team in case of need during the patient positioning and procedure.

An additional advantage of supine PCNL under RA is that it reduces the risk of thrombo-embolism secondary to the lack of IVC compressions [6,13], which facilitates maintaining the intrarenal pressure low as the access sheath is always either parallel to the floor or angulated downward facing the floor, which is contrary to what we do in the prone position. This helps in the spontaneous and effective drainage of the irrigating fluid with fragments and lowers the risk of infection compared to prone PCNL [14].

The mean fluoroscopic time in our study was 130 ± 60 s. Supine PCNL is associated with less radiation exposure compared to prone PCNL. This is attributable to the fact that during the puncture in case of supine PCNL, the hands are away from direct exposure [7], although no comparative data has yet been published.

PCNL under regional anesthesia has been found to be safe in all age groups [15,16]. Under GA, there is a risk of shoulder dislocation and neurological complications during the repositioning of the patient from supine to prone. Performing the procedure under RA averts this risk. At our center, we routinely perform prone PCNL under RA, particularly in elderly patients with co-morbidities to avoid the side effects of GA [16]. Therefore, we started performing the supine PCNL procedure in the modified Valdivia-Galdakao position also under RA.

The mean operative time in our study was 42 ± 10.3 min. The short operative time is explained by the fact that none of our patients required conversion to GA. The mean stone size in this series was 2.43 ± 0.5 cm. In the study by Mulay et al. [17], the mean operative time for similar size stones (2.43 cm) was 51 min. The shorter operative time in our series is likely attributable to the fact that we only included cases with a single pelvic stone.

Three patients in our study developed postoperative mild persistent hematuria and two patients reported postoperative fever. Both these complications responded to conservative treatment. Supine PCNL leads to effective drainage of irrigating fluid which decreases the risk of postoperative infection [14]. There was no adjacent organ injury or urosepsis in our series. Blood transfusion was required in only one patient. The mean drop in hemoglobin in our study (0.49 ± 0.43 mg/dL) was similar to that reported by Mulay et al. (0.37 g/dL) [17]. In their study, two out of the 50 patients (4%) undergoing supine PCNL developed postoperative fever. In contrast, 10% of patients in the study by Gutierrez et al. [18] developed fever despite taking antibiotics; this was attributed to predisposing factors such as pre-placed nephrostomy, staghorn calculus, positive urine culture, and comorbid diabetes. None of the patients in our series had these risk factors.

Postoperative pain VAS score was <5 in 31 (89%) patients and >5 in four (11%) patients. This was similar to the study by Mulay et al. in which postoperative pain VAS score was >5 in 12% of the patients [17]. This can be attributed to the prolonged analgesic effect of RA. The mean hospital stay in our study (3.33 ± 0.88 days) was similar to that reported by Gupta and Mahajan and Choudhury et al. (4.1 days) [16,19].

We were able to achieve 100% stone clearance in all cases, which was confirmed by postoperative X-ray and ultrasound. Our results are comparable with those reported by Gupta et al. In their five-year study on supine PCNL, they reported a stone-free rate of 92.4% [10]. Our better clearance rates may be attributed to the selection bias as only patients with a solitary pelvic stone were included.

The type of anesthesia (GA vs SA) or the position (supine vs prone) does not affect the clearance rates. This was studied by Gupta and Mahajan in elderly patients undergoing PCNL and they found no difference in the clearance rates in either group [16]. A meta-analysis by Wu et al. reported similar clearance rates for supine vs prone PCNL [20].

Morsy et al. [21] investigated the feasibility and safety of supine PCNL under RA in obese patients. They concluded that RA is feasible and safe in these patients with satisfactory stone-free rates and minimal postoperative pain. Although our study was done on patients having normal BMI, but we also achieved a 100% stone clearance rate with a mean VAS score < 5 in 89% of patients.

Study limitations

This was a retrospective, single-center study with small sample size. Moreover, we only included patients with solitary pelvic stone. With increasing experience, further study is required to assess the feasibility of supine PCNL for complex stones under RA. Secondly, we did not include patients with ASA PS > II. Supine PCNL under RA may be beneficial in such high-risk patients too. Future studies should include high-risk patients to gauge the benefit of this approach.

## Conclusions

We conclude that supine PCNL can be safely and effectively performed under RA, thereby avoiding the adverse effects of GA, with good stone clearance rates and minimal complications. The supine position also saves the overall operation time as preparing the supine PCNL position is always faster and easier than turning the patient prone. Moreover, patients under RA are responsive to the commands of the surgeon, thus creating harmony between the surgeon, anesthetist, and the patient.

## References

[1] Fernström I, Johansson B (1976). Percutaneous pyelolithotomy. A new extraction technique. Scand J Urol Nephrol.

[2] Alken P, Hutschenreiter G, Gunther R, Marberger M (1981). Percutaneous stone manipulation. J Urol.

[3] Smith AD, Lee WJ (1983). Percutaneous stone removal procedures including irrigation. Urol Clin North Am.

[4] Segura JW, Patterson DE, LeRoy AJ (1985). Percutaneous removal of kidney stones: review of 1,000 cases. J Urol.

[5] Macchi V, Picardi E, Inferrera A (2018). Anatomic and radiologic study of renal avascular plane (Brödel's line) and its potential relevance on percutaneous and surgical approaches to the kidney. J Endourol.

[6] Valdivia Urìa JG, Lachares Santamaría E, Villarroya Rodríguez S, Taberner Llop J, Abril Baquero G, Aranda Lassa JM (1987). Percutaneous nephrolithectomy: simplified technic (preliminary report). Arch Esp Urol.

[7] Proietti S, Rodríguez-Socarrás ME, Eisner B (2019). Supine percutaneous nephrolithotomy: tips and tricks. Transl Androl Urol.

[8] Hu H, Qin B, He D (2015). Regional versus general anesthesia for percutaneous nephrolithotomy: a meta-analysis. PLoS One.

[9] Chen M, Wu X, Zhang J, Dong E (2021). Prediction of total hospital expenses of patients undergoing breast cancer surgery in Shanghai, China by comparing three models. BMC Health Serv Res.

[10] Gupta S, Maurya AK, Pal DK (2019). Observational prospective study for surgical outcome and anesthetic feasibility of tubeless and totally tubeless supine PCNL: a single centre initial experience. Turk J Urol.

[11] Galvin DJ, Pearle MS (2006). The contemporary management of renal and ureteric calculi. BJU Int.

[12] Awan AS, Khalid S, Khan SA, Mithani S, Shaikh J, Sharif I (2019). Supine PCNL is the way forward, with reduced anesthesia and operative times as compared to prone PCNL, along with comparable blood loss and stone free rates. J Urol Surg.

[13] Feix B, Sturgess J (2014). Anaesthesia in the prone position. Continuing Educ Anaesth Crit Care Pain.

[14] Negrete-Pulido OR, Gutiérrez-Aceves J (2014). Antibiotic prophylaxis and infectious complications in PNL. In: Supine Percutaneous Nephrolithotomy and ERICS.

[15] Liu X, Huang G, Zhong R, Hu S, Deng R (2018). Comparison of percutaneous nephrolithotomy under regional versus general anesthesia: a meta-analysis of randomized controlled trials. Urol Int.

[16] Gupta R, Mahajan A (2020). Outcomes of percutaneous nephrolithotomy in elderly versus young patients under regional anesthesia: a comparative study. Urol Ann.

[17] Mulay A, Mane D, Mhaske S, Shah AS, Krishnappa D, Sabale V (2022). Supine versus prone percutaneous nephrolithotomy for renal calculi: our experience. Curr Urol.

[18] Gutierrez J, Smith A, Geavlete P (2013). Urinary tract infections and post-operative fever in percutaneous nephrolithotomy. World J Urol.

[19] Choudhury S, Talukdar P, Mandal TK, Majhi TK (2021). Supine versus prone PCNL in lower calyceal stone: comparative study in a tertiary care center. Urologia.

[20] Wu P, Wang L, Wang K (2011). Supine versus prone position in percutaneous nephrolithotomy for kidney calculi: a meta-analysis. Int Urol Nephrol.

[21] Morsy SM, Abdelaziz IN, Rammah AM, Labana MA, Hussein HA (2022). A prospective, observational study to assess the feasibility and safety of supine percutaneous nephrolithotomy under regional anesthesia for obese patients with a body mass index ≥30. Indian J Urol.

